# *In vitro* analyses of *Artemisia* extracts on *Plasmodium falciparum* suggest a complex antimalarial effect

**DOI:** 10.1371/journal.pone.0240874

**Published:** 2021-03-02

**Authors:** Brian M. Gruessner, Pamela J. Weathers

**Affiliations:** Department of Biology and Biotechnology, Worcester Polytechnic Institute, Worcester, Massachusetts, United States of America; Instituto Rene Rachou, BRAZIL

## Abstract

Dried-leaf *Artemisia annua* L. (DLA) antimalarial therapy was shown effective in prior animal and human studies, but little is known about its mechanism of action. Here IC50s and ring-stage assays (RSAs) were used to compare extracts of *A*. *annua* (DLAe) to artemisinin (ART) and its derivatives in their ability to inhibit and kill *Plasmodium falciparum* strains 3D7, MRA1252, MRA1240, Cam3.11 and Cam3.11rev *in vitro*. Strains were sorbitol and Percoll synchronized to enrich for ring-stage parasites that were treated with hot water, methanol and dichloromethane extracts of DLA, artemisinin, CoArtem™, and dihydroartemisinin. Extracts of *A*. *afra* SEN were also tested. There was a correlation between ART concentration and inhibition of parasite growth. Although at 6 hr drug incubation, the RSAs for Cam3.11rev showed DLA and ART were less effective than high dose CoArtem™, 8 and 24 hr incubations yielded equivalent antiparasitic results. For Cam3.11, drug incubation time had no effect. DLAe was more effective on resistant MRA-1240 than on the sensitive MRA-1252 strain. Because results were not as robust as observed in animal and human studies, a host interaction was suspected, so sera collected from adult and pediatric Kenyan malaria patients was used in RSA inhibition experiments and compared to sera from adults naïve to the disease. The sera from both age groups of malaria patients inhibited parasite growth ≥ 70% after treatment with DLAe and compared to malaria naïve subjects suggesting some host interaction with DLA. The discrepancy between these data and *in-vivo* reports suggested that DLA’s effects require an interaction with the host to unlock their potential as an antimalarial therapy. Although we showed there are serum-based host effects that can kill up to 95% of parasites *in vitro*, it remains unclear how or if they play a role *in vivo*. These results further our understanding of how DLAe works against the malaria parasite *in vitro*.

## Introduction

In 1972 artemisinin (ART) was discovered as an antimalarial compound in ethyl-ether extracts of *Artemisia annua* L., or sweet wormwood [1]. Since then, ART and its derivatives have become critical in treatment regimens for malaria around the world. Conventionally, ART is combined with a partner drug to create an artemisinin combination therapy (ACT) [2,3]. The rationale behind these combination drugs was that it would prevent the emergence of drug resistance against either of the components of the ACTs.

Despite these precautions, ART drug resistance [4–6] and reduced efficacy of ACTs [7,8] have emerged in Southeast Asia. There is concern that these resistant strains will spread to Africa, where >90% of fatalities from malaria occur [9], and may indeed now be occurring [10]. While ACTs remain generally effective in Africa [2,11], there have been isolated reports of slow parasite clearance [12] and ACT failures [13] in the region. There is an urgent need for a new treatment for malaria before ART resistance becomes pandemic. If ACTs were to become obsolete without a suitable backup, it is estimated that an additional 116,000 annual deaths will occur from the disease [14].

The use of the dried leaves of *A*. *annua* (DLA) is a possible alternative to conventional ACTs. In mouse models of malaria, DLA substantially outperformed relatively higher doses of ART in suppressing parasitemia [15]. DLA also hindered development of resistance from repeated exposure compared to ART at the same amount in the murine *P*. *yoelli* malaria model [16]. In one case study it successfully treated patients that did not respond to treatments with ACTs and intravenous artesunate [17]. Despite WHO cautions against using *Artemisia* sp. [3], there is evidence demonstrating that DLA exerts antimalarial activity well beyond its ART content and *in vitro* assays could help explain this alternative therapeutic approach.

Understanding the mechanisms behind the enhanced activity of DLA also would have important ramifications for its medicinal acceptance and deployment. However, these mechanisms are not well-established. *Artemisia* extracts contain dozens of other phytochemicals with, albeit weak, antimalarial activity [18–21]. In some cases, these phytochemicals can add to or even synergize with the plant’s ART [20]. However, there are other potential mechanisms. Among these are the improvement in the apparent pharmacological properties of ART when consumed in leaf form, as observed in mice [22]. This activity could stem from improved aqueous solubility of ART in extracts and teas [23], likely due to emulsification in amphiphilic compounds from *A*. *annua* such as essential oils [24,25]. Compared to the pure drug, ART delivered in dried-leaf form is also more efficiently transported across the intestinal wall in an *in-vitro* Caco2 and simulated digestion model [26]. Additionally, flavonoids that are known components of *A*. *annua* extracts inhibit cytochrome P-450 liver metabolic enzymes known to degrade ART, which would improve the drug’s biological persistence when delivered in a DLA-derived form [27]. DLA anti-inflammatory effects [28] and effects on helper T-cells [29] could also be relevant to its antimalarial effects. Indeed, DLAe inhibited both CYP2B6 and 3A4 in human liver microsomes and DLA dampens inflammation markers in a rat model [30].

There is no apparent consensus on whether extracts of *A*. *annua* exhibit a decreased IC50 then what would be predicted only by their ART content. Different *in-vitro* studies have concluded that the tea of *A*. *annua* shows at least a threefold decrease in IC50 compared to its ART content [20,31] and that the IC50 of *A*. *annua* teas are entirely explained by their ART content [32,33]. Recently, Czechowski *et al*. (2019) [34] used an *A*. *annua* mutant with normal levels of flavonoids that produces no ART to challenge *P*. *falciparum in vitro*. They attributed all activity to only the presence of ART and not the presence of antimalarial flavonoids. In contrast, Snider and Weathers (2020) [35] showed inhibition of trophozoites by *A*. *afra* tea infusions that contained no detectable ART.

This report examines the possibility that components in *A*. *annua* extracts synergize with ART and decrease the IC50 of the extracts compared to the IC50 of pure ART. While it was hypothesized that the extracts would outperform ARTs or ACTs in their ability to inhibit plasmodia growth and survival, this effect was not clearly observed. Instead, this report suggests a DLA mechanism of antimalarial activity may involve a host-DLA interaction.

## Materials and methods

### Chemical stocks

Dihydroartemisinin (DHA) and ART were formulated to 700mM in DMSO. Dihydroartemisinin-piperaquine (DHAPPQ) was formulated to 1:8 DHA:PPQ w/w to working concentration of 700nM DHA. CoArtem^TM^ (6:1 (w/w) artemether + lumefantrine) was formulated to 700mM in DMSO. All stocks were aliquoted after formulation and frozen at -20°C. From these concentrations, the CoArtem^TM^ and DHAPPQ could be diluted 1000X in media to working concentrations.

### Plant material

*Artemisia annua* L. SAM cultivar (harvested 2014, voucher MASS 00317314) was cultivated in Stow, MA, USA, 42.4370° N, 71.5056° W and leaves harvested and dried as described in Weathers and Towler 2014 [18]. The dried leaves (DLA) contained 15.9 mg/g DW ART and 2.8 mg/g DW total flavonoids; a more detailed phytochemical content is provided in Weathers and Towler 2014 [18]. *A*. *afra* (voucher LG0019529, from Guy Mergeai, Université de Liege) was the SEN cultivar grown in Senegal at the Ecole National Supérieure d’Agriculture de Thiès (ENSA-Université de Thiès) 14.7656° N, 16.8911° W. The phytochemical content of SEN *A*. *afra* contains negligible ART (0.037 mg/g DW), but has a total flavonoid content of 2.03 mg/g DW. Both Artemisia species were harvested in their vegetative phase, prior to anthesis. Table 1 outlines the properties of the cultivar extracts used in this study.

**Table 1:** Artemisinin and flavonoid content and mean IC50s vs. *3D7 P*. *falciparum* of *Artemisia* extracts.

Species	Cultivar	Extract	ART (mg/g DW)	IC50 (μg ART/L)	IC50 (nM ART)	FLV (mg/g DW)	IC50 (μg FLV/L)	IC50 (μg DW/L)
N/A	N/A	Artemisinin	N/A	9.4 (2.4–15.3)	26.6 (6.8–43.1)	N/A	N/A	N/A
*A*. *annua*	SAM	Dichloromethane	9.44	3.79	13.4	2.05	1.00	401.35
*A*. *annua*	SAM	Methanol	8.94	3.00	10.6	6.39	2.14	335.47
*A*. *annua*	SAM	Tea	15.98	7.21	25.5	2.46	1.11	451.36
*A*. *annua*	SAM	Combo	11.45	8.06	28.5	3.63	2.54	701.17
*A*. *afra*	SEN	Dichloromethane	0.04	6.58	23.3	2.03	3,610.65	177,864.26

ART, artemisinin; Combo, a 1:1:1 mixture of the dichloromethane, methanol, and tea extracts; DW, dry weight of *Artemisia* leaves; FLV, flavonoids.

### Extracts

Dried leaves of each plant species were ground in a Kitchen Aide coffee grinder for 3, 5 sec pulses with a cube of dry ice. For each extraction, 6 g of the ground leaves were then divided into 6, 1 g portions and each was extracted three times with 20 mL dichloromethane or methanol for 30 min in a sonicating water bath. Extracts were pooled and filtered through a pipet stuffed with glass wool to remove solid debris. Aliquots of dichloromethane and methanol extracts were analyzed for flavonoid content via an aluminum chloride assay adapted from Arvouet-Grand et al. [36]. Briefly, extracts of *A*. *annua* equivalent to 1mg plant material were resuspended to 1mL in 1% aluminum-chloride in methanol. Following a 30 min room-temperature incubation, absorbance was measured at 415 nm, using quercetin in a standard curve with total flavonoids expressed in quercetin units. Another aliquot of each extract in each solvent was analyzed for ART via gas chromatography-mass spectroscopy as detailed by Martini et al. [37]. Using these ART concentrations, the remaining bulk of the extract was dried and resuspended to 20 mM ART in DMSO. A dichloromethane *A*. *afra* extract was similarly prepared and suspended such that its ART content was 335 μM ART in DMSO (equivalent to 2,100 mg plant DW/mL).

For the *A*. *annua* tea, 5 g of leaves were steeped in 1 L of boiled water for 10 min and then filtered sequentially through a 1 mm sieve, a 600 μm sieve, Whatman #1 filter paper, a nitrocellulose filter, a 0.45 μm Millipore type HA filter, and a sterile 0.22 μm cellulose acetate bottle top filter (Corning). The tea was then aliquoted into small vials and frozen at -80° C.

For the “combined” *A*. *annua* extract, the dichloromethane, methanol, and tea extracts were mixed to the same concentration of ART in solvent (DMSO for the dichloromethane and methanol extracts, water for the tea extract) and combined in equal volumes. This combination was then added to complete media and DMSO to final working concentrations of 0.16–100 nM artemisinin content in 0.1% DMSO.

### Plasmodia culturing

Plasmodia (strains 3D7, MRA1252, MRA1240) were from BEI Resources. The sensitive/resistant strain pair Cam3.II *R539T wt revertant* was a generous gift from David Fidock at Columbia University. All strains are shown in Table 2, stored in liquid nitrogen and thawed as per [38]. To measure the IC_50_, the plasmodia were cultured with type A+ red blood cells (RBCs; Red Cross) at 4% hematocrit and 1–5% parasitemia. For other experiments, the conditions were 5% hematocrit and 1–5% parasitemia. The cells were incubated in Complete Malaria Media adapted from [39] containing 10% Human Serum (SHS; Sigma), 10.4 g/L RPMI, 5.94 g/L HEPES, 4 g/L glucose, 44 mg hypoxanthine in 1 mL sodium hydroxide/L, 4mg/mL gentamycin, adjusted to pH 7.4 with 5% sodium bicarbonate. Cultures were kept in 12.5 cm^2^ tissue culture flasks at 37°C in 90% N_2_, 5% CO_2_, 5% O_2_ and split to 25 cm^2^ tissue culture flasks for experiments. Culture medium was changed daily, and red blood cells were replaced every other day.

**Table 2:** *Plasmodium falciparum* strains used in this study.

Strain	Source and strain notes
3D7	BEI Resources MR-102; CQ sensitive
Cam 3.II	Fidock lab; K13 mutant
Cam 3.IIrev	Fidock lab; K13 revertant to wt
Cam3.I	BEI Resources MRA 1240; AN resistant; probable mutation in wt allele R539T
Cam3.Irev	BEI Resources MRA 1252; K13 reversion in wt allele R539T; no longer AN resistant

### IC50s of artemisinin and DLA extracts

To measure the IC50, cultures of 3D7 and the ART resistant/sensitive revertant strain pair MRA1240/MRA1252 (also known as Cam3.I/Cam3.Irev) were synchronized with 2, 15 min, 5% sorbitol incubations, 48 hr apart. Cultures, 100 μL each, were incubated in serial dilutions of either CoArtem^TM^ or extracts of *A*. *annua* in 96-well plates for 72 hr [39]. RBCs infected with 3D7 were synchronized via incubation in five pellet volumes of 5% sorbitol for 15 min and incubated in serial dilutions of ART paired with the following: *A*. *annua* dichloromethane or methanol extract, *A*. *annua* tea, a combination of the three (*A*. *annua* dichloromethane extract, methanol extract, and tea*)*, or *A*. *afra* dichloromethane extract, in 0.1% DMSO for 72 hrs. In each experiment, the cells were also incubated in 0.1% DMSO and the CoArtem^TM^ control as references.

### Determination of final relative parasitemia

Final relative parasite growth for all IC50 determinations, ring-stage survival assays (RSAs), and serum inhibition experiments were derived using a SYBR Green-based fluorometric method from Johnson et al. 2007 [39]. Briefly, after the incubation periods for the growth inhibition and ring-stage survival assays, the 96-well plates were frozen at -80°C at least overnight. The plates were thawed with 100 μL of 2X lysis buffer containing SYBR Green for three hours, with brief intermittent shaking on a plate shaker at 0, 1.5, and 3 hours and then read on a fluorescence plate reader at 485 nm excitation and 535 nm emission wavelengths.

### Ring-stage survival assays

The RSA synchronization and drug/extract treatment protocol was that of Straimer et al 2015 [40] with the following adaptations. Cultures were synchronized with an initial double sorbitol synchronization as previously described, and then further synchronized with a Percoll column schizont enrichment 38 hr after the second sorbitol synchronization. After allowing 3 hr for reinvasion, a final sorbitol synchronization was performed before incorporation into the experiment.

In the initial RSAs, the MRA1252/MRA1240 (Cam3.I/Cam3.Irev) strain pair was used. Infected RBCs were treated with 0.1% DMSO, 700 nM artemether+ 2,366 nM lumefantrine (maintained for full 72 hr) in 0.1% DMSO, 700 nM dihydroartemisinin (DHA) in 0.1% DMSO, and dichloromethane and methanol extracts to 700 nM ART in 0.1% DMSO. The strain pair was incubated with these drug treatments at 1% hematocrit, 1% parasitemia, to 150 μL per treatment in 1.5 mL tubes, with each treatment in triplicate. This incubation was performed at 37°C, in the above gas environment, for 6 hr. Each culture was then washed twice with 1mL Complete Malaria Media with a final resuspension of the pellet to 150 μL with Complete Media (the CoArtem^TM^ control was resuspended in CoArtem^TM^ to 700 nM artemether). 100 μL aliquots of the treatments were then added to a 96-well plate to incubate for an additional 66 hr.

In the RSA time-course experiments, the Cam3.II/Cam3.IIrev strain pair was used. This strain pair differs in a single *kelch13* SNP that produces the ART-resistance-inducing R539T mutation. In each trial, the conditions used were ART in 0.1% DMSO, methylene-chloride extract of *A*. *annua* in 0.1% DMSO, and methanol extract of *A*. *annua* in 0.1% DMSO to 70 nM ART, 70 nM DHA in 0.1% DMSO, CoArtem^TM^ to 700 nM artemether in 0.1% DMSO (maintained for the full 72 hr), and 0.1% DMSO in media were used in the experiment. Incubations were performed for 6, 8, and 24 hr before washout in 96-well plates.

### Sera comparison ring-stage growth inhibition assays

RBCs infected with the 3D7 strain of *P*. *falciparum* were synchronized to the ring stage as previously described then combined with serum in 96-well plates to a working concentration of 10% culture volume. The pooled serum samples (from 7–12 individuals for each test group), courtesy of Prof. Ann Moorman University of Massachusetts Medical School (IRB approvals: University of Massachusetts Medical School, UMMS H00004587_12; Kenya Medical Research Institute, KEMRI SSC 2844;), came from either malaria-naive US adults (US), malaria-infected Kenyan adults (KA), or malaria-infected Kenyan pediatrics (KP). Data were from pooled samples and analyzed anonymously. To probe for interactions between antimalarial treatments and components of the immune system that had recent exposure to malaria vs. no exposure to malaria, cultures were treated with various drugs/extracts including ART, a dichloromethane extract of *A*. *annua* (DCM), and a tea infusion preparation of *A*. *annua* cv. SAM, DMSO as a negative solvent control, and DHAPPQ to 700nM DHA, as a total growth-inhibition control. Although at high concentrations (700 M) CoArtem™ and DHAPPQ are both suitable positive-kill controls, DHAPPQ is the more clinically relevant ACT to use for Kenyan patient samples. Plates were incubated under parasite culturing conditions for 72 hr. Subsequent 72-hr growth inhibition assays had the following change. Serum treatments were diluted 1:1 with Sigma-Aldrich human serum (SHS; cat #H4522) before incorporation into the experiment. All drug treatments were performed at 9, 18, and 36 nM ART concentrations.

### Serum concentration calibration

Treatment sera were diluted with SHS to 100%, 80%, 60%, 40%, and 20% treatment serum concentrations and then incorporated into a 72-hr growth assay as previously described to a working concentration of 10% total human serum. SHS, and DHAPPQ treatment in SHS were controls.

### Serum supplementation experiments

Seven serum groups were assayed for growth by 72-hr incubation and SYBR assay as previously described: 10% SHS, 10% SHS + DHAPPQ (as previously described), 10% SHS + 10% water, 20% SHS, 10% SHS + 10% Kenyan Adult serum, 10% SHS + 10% Kenyan Pediatric serum, and 10% SHS + 10% US Adult serum.

### Statistics

All statistical analyses were performed using GraphPad Prism 8. IC50s of drugs and extracts were derived from the inhibition data using GraphPad and a 4P Sigmoidal Curve fit. While fitting to a sigmoidal model, the maximum value was set to the fluorescence value of untreated control and the minimum value set to the value of the CoArtem^TM^ control. The derived ART IC50 of a given extract treatment was divided by the IC50 of the ART group run on the same plate to yield a -fold decrease in apparent IC50 of the extract(s) compared to ART. From ≥3 replicate trials of these experiments for each extract group, the geometric mean of the -fold change metrics were used and compared. Extract groups that suggested enhanced activity vs. ART alone (>1.5-fold change) or that were otherwise of interest were further repeated to statistically validate the -fold change. For groups with enough replicates for statistical analysis, confidence intervals and one-sample two-tailed t-tests were performed on the log_2_ transformed ratios to determine whether there was a statistically significant difference between the IC50s of the plant extracts and ART (H_0_: log_2_(IC50_ART_/IC50_extract_) = 0, H_A_: log_2_(IC50_ART_/IC50_extract_) ≠ 0). The α value for this experiment was adjusted to account for the five extracts (Table 1) compared with ART (α = 0.05/5 = 0.01). Log-log plots of the extracts showing IC50s in μg dry weight of plant material (DW)/L vs the flavonoid and ART content were produced. Linear regressions, R^2^ values, and P values of the slope being zero were derived for both regressions to determine correlation between phytochemical content and antimalarial potential.

For the RSAs performed with the Cam3.I/Cam3.Irev strains, there were six biological replicates of experiments, each with three technical replicates. The non-DMSO groups within each strain were compared using a Friedman test. Pairwise comparisons of all pairs with Dunn’s correction were then performed. For the RSAs performed with the Cam3.II/Cam3.Iirev strains, there were six biological replicates of experiments, each with three technical replicates. For statistical analysis, the non-DMSO control treatments within each strain and time-point were cross-compared using a repeated measures one-way ANOVA. Pairwise comparisons of all pairs with Tukey’s correction were then performed.

IC50s for the serum comparison ring-stage inhibition assays were performed by identifying the two points between which was the IC50, and extrapolating a linear curve between the two points to calculate the IC50. For curves in which the curve did not cross the IC50 point or crossed twice, the replicate was excluded from analysis.

## Results

Overall, the extracts of *A*. *annua* had ~9–16 mg ART/g dry *Artemisia* leaf mass (DW) (Table 1). The dichloromethane extract had an ART content of 9.44 mg/g DW, compared to the methanol extract’s 8.94 mg/g DW and the tea’s 15.98 mg/g DW. However, *A*. *afra* had a much lower content of ART (0.04mg/g DW). The dichloromethane extracts of *A*. *annua* and *A*. *afra* had similar flavonoid content, at 2.05 and 2.03 mg flavonoids/g DW, respectively. In contrast, the methanol extract of *A*. *annua* had a much higher flavonoid content at 6.39mg/g DW. The *A*. *annua* tea had a flavonoid concentration similar to the DCM extracts, at 2.46 mg/g DW. The combination extract had an ART content of 11.45 mg/g DW and a flavonoid concentration of 3.63 mg/g DW.

The derived IC50 of ART against the 3D7 strain across many trials was about 26.6 nM (9.4 μg/L). However, the IC50s derived from individual experiments were prone to considerable variation, ranging from 6.8–43.1 nM (2.4–15.3 μg/L). Comparisons of the relative IC50s between ART and extracts within a single experiment thus were used to compare the efficacy of the treatments. The values in Tables 3 and 4 reflect the quotient of the IC50 of ART divided by the IC50 of the *Artemisia* extract treatments run on the same plate, wherein values > 1 reflect potentiation of the inhibitory activity of the extracts.

**Table 3:** IC50s of ART of and various extracts. Experiments were performed using the 3D7 strain of *P*. *falciparum*. ART, artemisinin; DLA*e*, dried leaf *Artemisia* extract; Combo, a 1:1:1 mixture of the dichloromethane, methanol, and tea extracts.

Extract Type	*A*. *annua* Dichloro-methane DLA*e*	*A*. *annua* Methanol DLA*e*	*A*. *annua* Tea	*A*. *annua* Combo	*A*. *afra* Dichloro-methane DLA*e*
**Biological Replicates**	7	3	3	3	5
**Geometric-Mean IC50 nM ART/IC50 Extract (nM ART)**	20.9/12.3 = 1.70X	11.8/9.9 = 1.20X	30.2/23.6 = 1.28X	30.2/26.9 = 1.16X	26.0/22.1 = 1.18X
**Geometric-Mean IC50 μg/L ART/IC50 Extract (μg/L ART)**	5.9/3.5 = 1.70X	3.3/2.8 = 1.20X	8.5/6.7 = 1.28X	8.5/7.6 = 1.16X	7.3/6.2 = 1.18X
**Log(2) Mean**	0.77	0.26	0.36	0.22	0.24
**Std. Error of Log(2) Mean**	0.23	0.19	0.17	0.08	0.30

**Table 4:** Paired comparisons of the IC50s of ART and dichloromethane *Artemisia* extracts. Experiments performed using the Cam3.II/Cam3.II ART resistant/sensitive revertant strain of *P*. *falciparum*. ART, artemisinin; DCM, dichloromethane.

	Cam3.IIrev *A*. *annua*	Cam3.IIrev *A*. *afra*	Cam3.II *A*. *annua*	Cam3.II *A*. *afra*
**Biological Replicates**	2	2	2	2
**Geometric Mean IC50ART/IC50Extract (nM ART)**	17.3/15.9 = 1.09X	17.3/16.9 = 1.03X	19.2/19.9 = 0.97	19.2/22.4 = 0.86
**Geometric Mean IC50ART/IC50Extract**	4.9/4.5 = 1.09X	4.89/4.92 = 1.03X	5.4/5.6 = 0.97X	5.4/6.3 = .86X
**Log(2) Mean**	0.12	0.04	-0.05	-0.22

**Fig 1:**
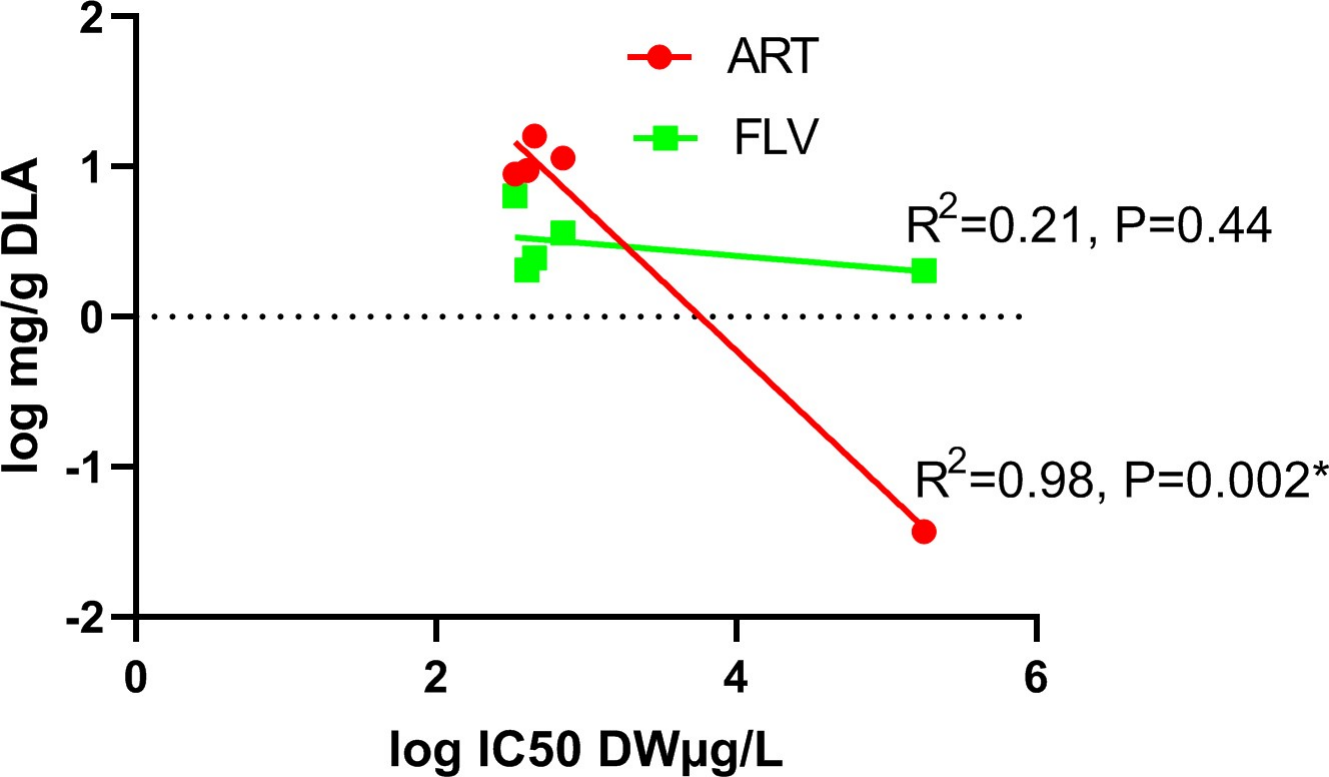
Artemisinin content, but not flavonoid content, correlates well with leaf extract IC50s. ART, artemisinin; DW, dry weight of Artemisia leaves; FLV, total flavonoids.

Regardless of this variation, the mean IC50s of the treatments are within an order of magnitude of each other when measured by artemisinin concentration (3.79–7.21 μg/L), but not flavonoid concentration (1.00–3610.65 μg/L) or by overall plant DW (335.47–177,864.26 μg/L). There was a strong correlation between the ART concentration of the extracts and their potential to inhibit plasmodial growth (Fig 1). The R^2^ of the linear regression of this relationship was 0.98 and its slope is significant (P = 0.002, α = 0.05/2 = 0.025). In contrast, the relationship between flavonoid content and antimalarial potential was weak (R = 0.21) and the slope of the linear regression of the relationship was not significant (P = 0.44, α = 0.05/2 = 0.025).

### IC50s and synergy of components of DLA extracts

**Fig 2:**
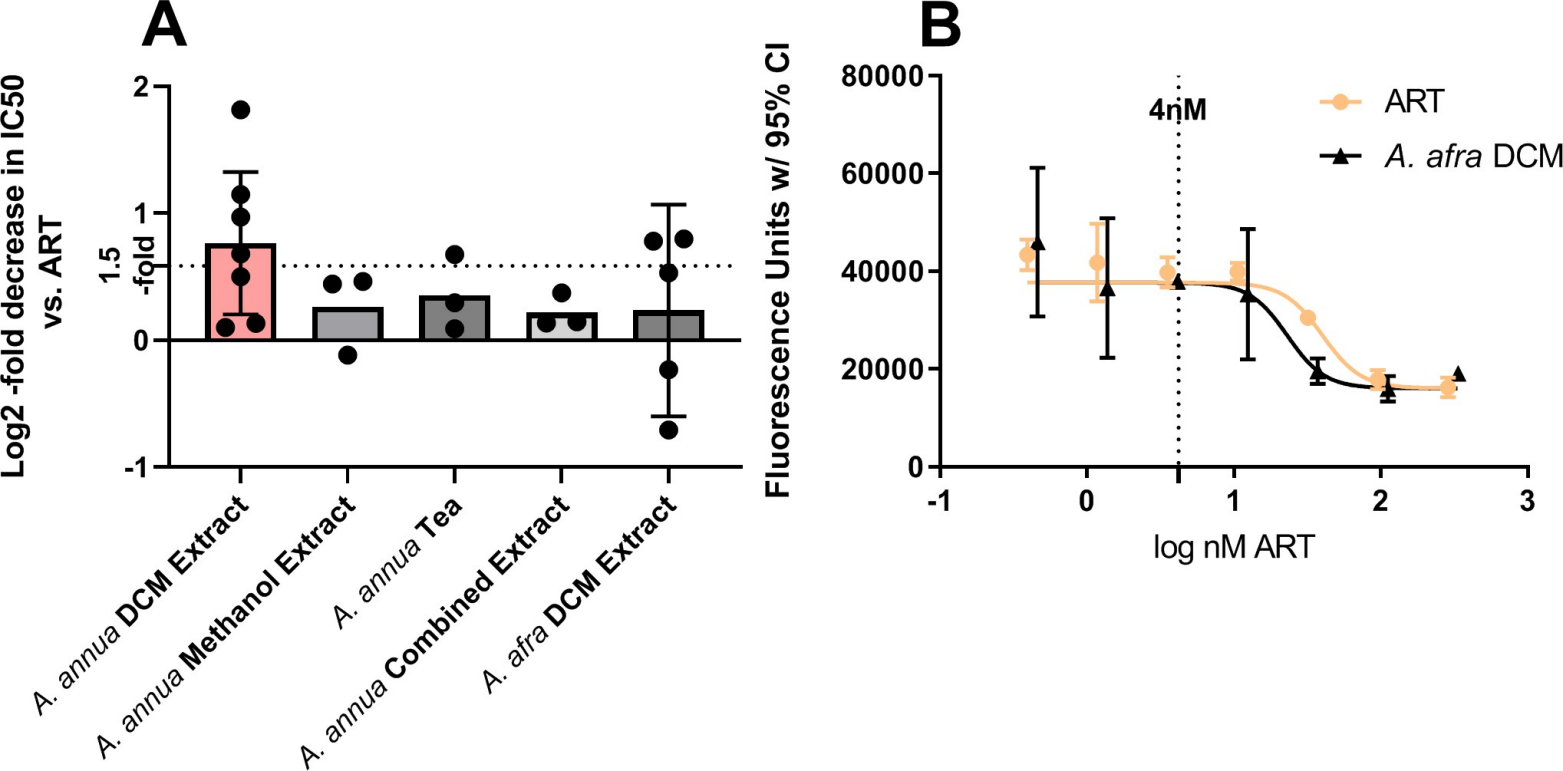
Relative IC50s of various extracts compared to ART against *Plasmodium falciparum*. A, various extracts vs. 3D7. B, Sample inhibition curves of ART and DLAe of *A*. *afra* against 3D7. Comparisons were made in the log2 scale wherein values >0 represent potentiation of antimalarial activity in extracts. α = 0.05/5 = 0.01. ART, artemisinin; DCM, dichloromethane; IC50, concentration of half-maximal inhibition.

To assess the ability of the extracts to inhibit plasmodial growth, cultures of plasmodia (3D7) were grown in the presence of varying concentrations of ART and *A*. *annua* extracts. An extract of the plants using dichloromethane, methanol, an aqueous tea, and a 1:1:1 combination of the three were performed. Additionally, a dichloromethane extract of *A*. *afra* was included in this study due to its antimalarial activity and because it contained little to no ART. Of these five extracts examined, only the dichloromethane extract of *A*. *annua* showed a >1.5-fold enhancement of activity following three replicates (Table 3, Fig 2A). After seven biological replicates, each of which had multiple technical replicates, the *A*. *annua* dichloromethane extract had a relative IC50 1.7-fold higher than ART alone (*p* = 0.021, α = 0.01). In contrast, the *A*. *afra* dichloromethane extract had a relative IC50, based on its ART content, 1.17-fold lower than ART alone (*p* = 0.34, α = 0.01, N = 5); see sample curves in Fig 2B.

**Fig 3:**
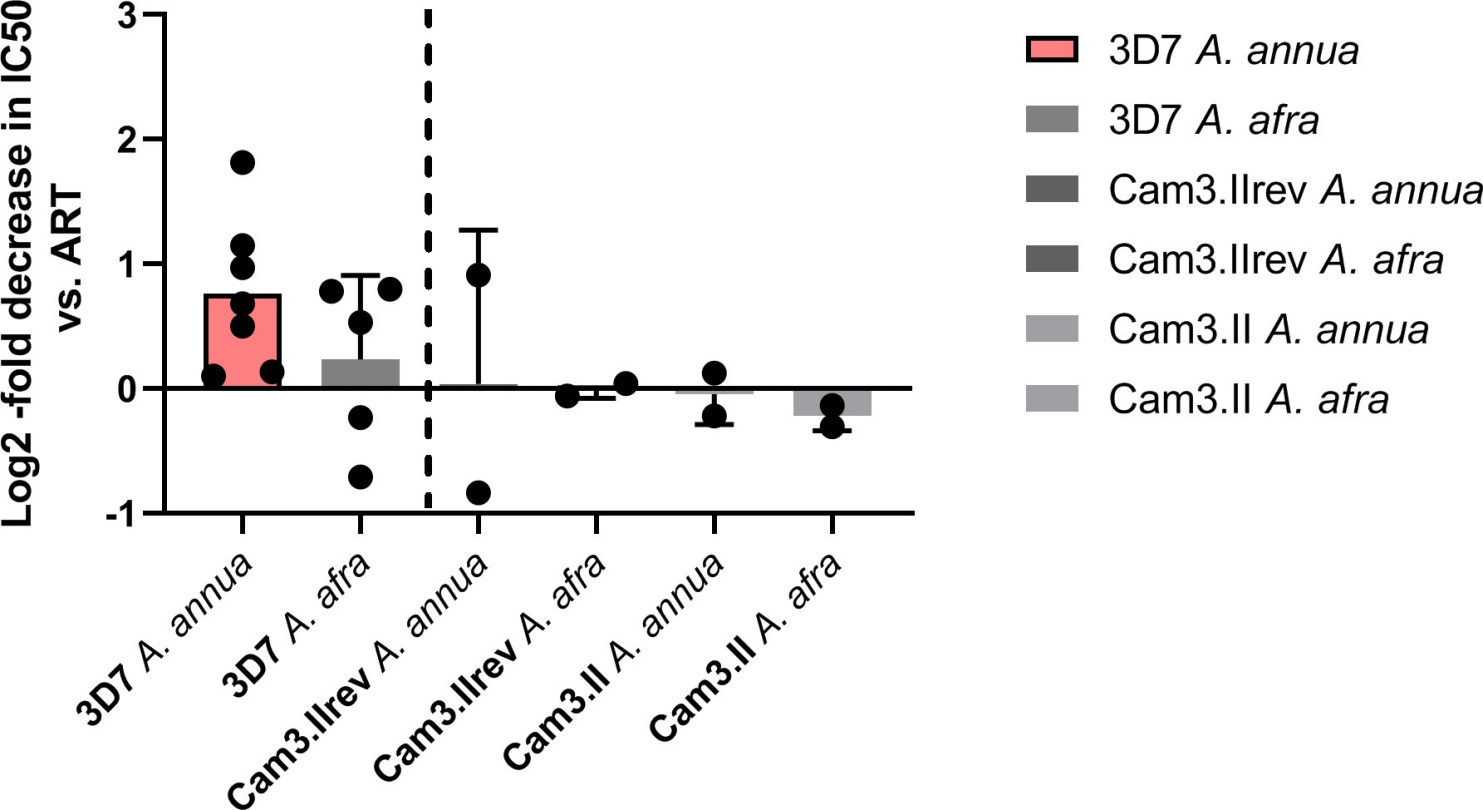
Relative IC50s of various dichloromethane extracts compared to ART. Comparisons were made in the log2 scale wherein values >0 represent potentiation of antimalarial activity in extracts. The left pair of bars are the 3D7 IC50 experiments from Fig 2, reprinted here for ease of comparison. ART, artemisinin; IC50, concentration of half-maximal inhibition.

The IC50 comparisons for the *A*. *annua* and *A*. *afra* dichloromethane extracts were repeated in the ART-resistant strain Cam3.IIR593T and its sensitive revertant Cam3.IIrev. In these experiments, the fold decrease in IC50 compared to ART alone was <1.03 for all comparisons (Table 4, Fig 3).

Overall, the dichloromethane extract of *A*. *annua* yielded an average IC50 of about 13.42 nM ART, or 0.40 μg plant DW/mL. *A*. *afra* had an average IC50 of 21.2 nM ART, or about 125.5 μg plant DW/mL. Although in a comparison of three trials in which the *A*. *annua* and *A*. *afra* dichloromethane extracts were run in parallel and the IC50 of *A*. *annua* seemed greater, they were within the 95% CI of each other (Table 4, Fig 2A). This is despite the orders of magnitude difference in ART content between the two species.

### No apparent phytochemical synergism vs. ring-stage

**Fig 4:**
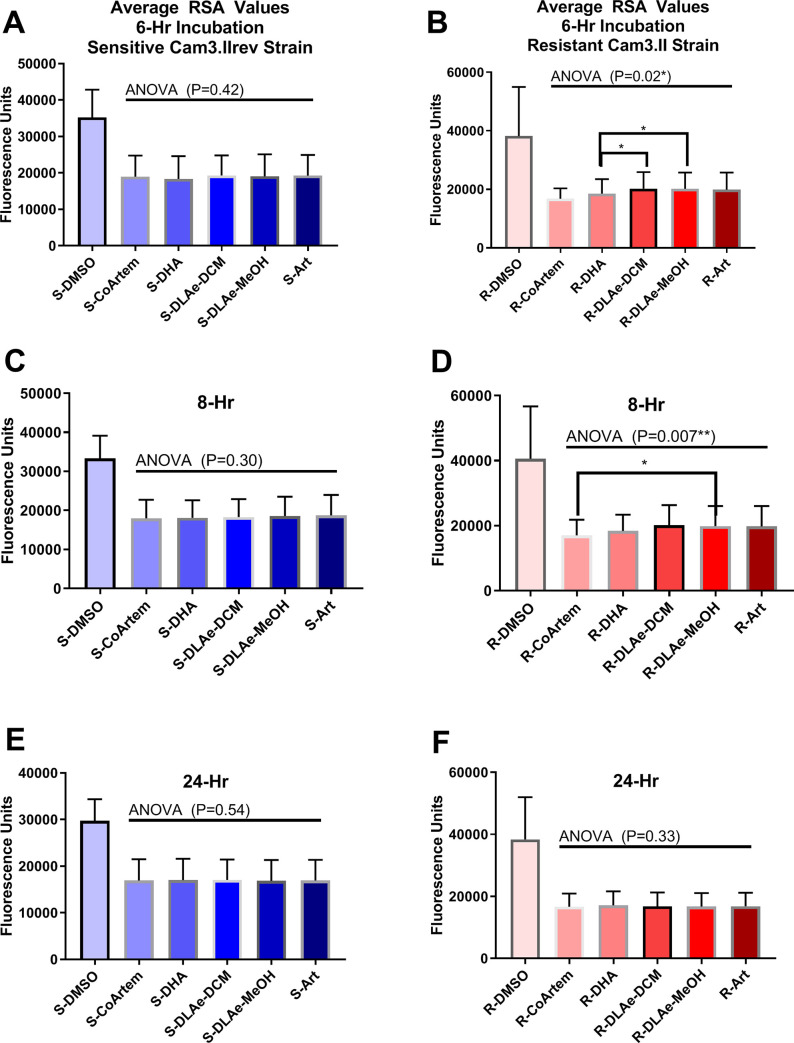
Results of 6, 8, and 24 hr RSAs in the (A,C,E) Cam3.IIrev, and (B,D,F) Cam3.II, strains. (N = 6). A,C,E: Cam3.IIrev strain, B, D, F: Cam3.II strain. ART, artemisinin; DCM, dichloromethane; DHA, dihydroartemisinin; DLAe, dried leaf *Artemisia annua* extract; MeOH, Methanol; R, resistant; S, sensitive. *: P<0.05, **: P<0.01.

To determine the degree of treatment killing efficacy, *P*. *falciparum* culture recovery following a brief drug treatment was measured using the ring-stage survival assay (RSA). After a 6-hr treatment, DHA, extracts, ART, and a high dose of CoArtem^TM^ were all statistically comparable treatments in an ART-sensitive strain (Fig 4A). However, in the ART-resistant Cam3.II strain, extracts of *A*. *annua* were significantly less effective than DHA (Fig 4B).

**Fig 5:**
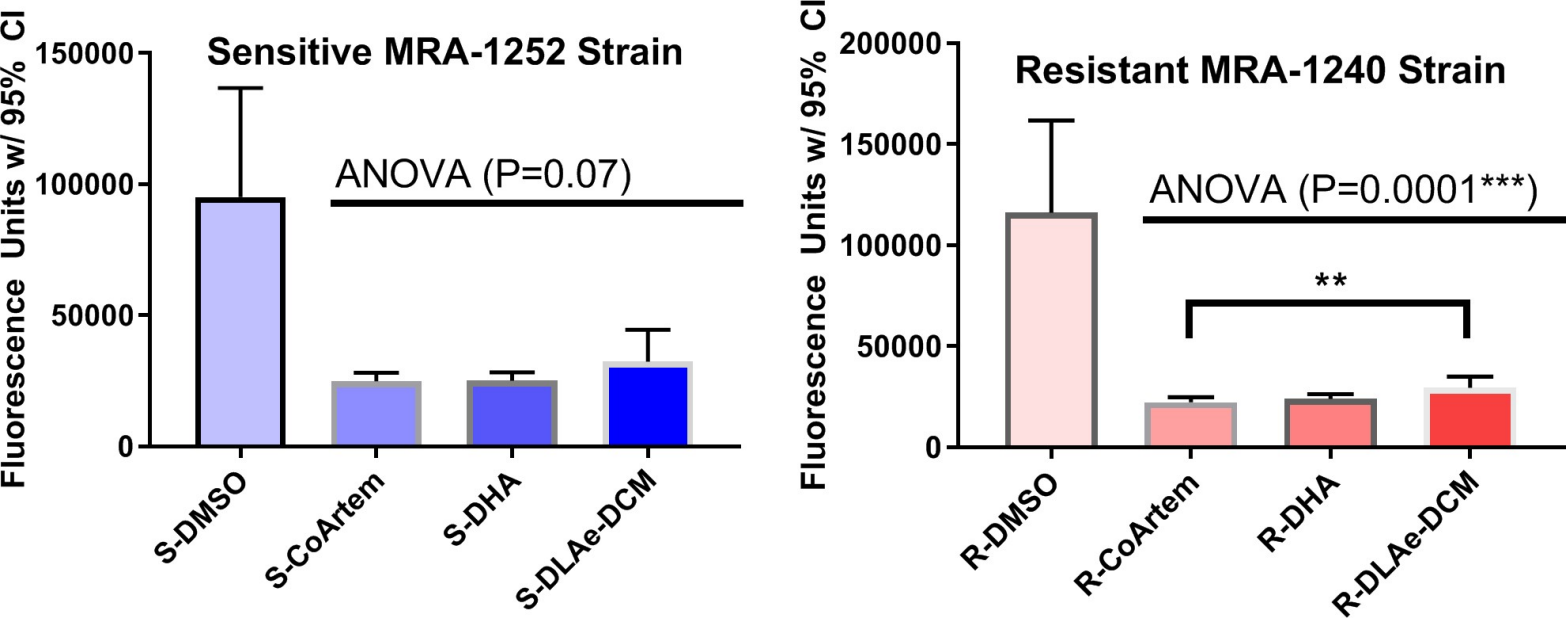
Results of 6-hr RSAs in the MRA-1252 and MRA-1240 strains (N = 5). DCM, dichloromethane; DHA, dihydroartemisinin; DLA*e*, dried-leaf *Artemisia* DCM extract. *: P<0.05, **: P<0.01, ***: P<0.001.

Analogous experiments performed with the MRA1240/MRA1252 strain pair revealed similar trends (Fig 5). In this experiment, a dichloromethane extract of *A*. *annua* on average yielded greater fluorescence values than the CoArtem^TM^ treatment. This result suggested there was detectable recovery from the extract treatment, while this was not true of the DHA treatment. The data from the Cam3.II and MRA1240/1252 strain pairs both challenge the notion that the extracts have superior *in-vitro* cytotoxic anti ring-stage activity to ART derivatives.

Following an 8-hour RSA, the relative effects of the treatments were similar, but the magnitude of the differences was less pronounced (Fig 4C and 4D). Again, the differences between the treatments in the Cam3.IIrev strain were not statistically significant. In the resistant strain, the CoArtem^TM^ control and the DHA treatments yielded significantly lower SYBR Green fluorescence than the methanol extract of *A*. *annua* (Fig 4D). There were no differences in plasmodial yield in either strain following 24-hour incubations in each treatment (Fig 4E and 4F). After 24 hours *in-vitro* incubation, all the treatments were effective, and the level of potential surviving parasites fell below the limit of detection of the assay; data not shown because post 24 h there were no further changes in parasite growth.

### Sera from malaria, but not malaria-naïve patients killed parasites

**Fig 6:**
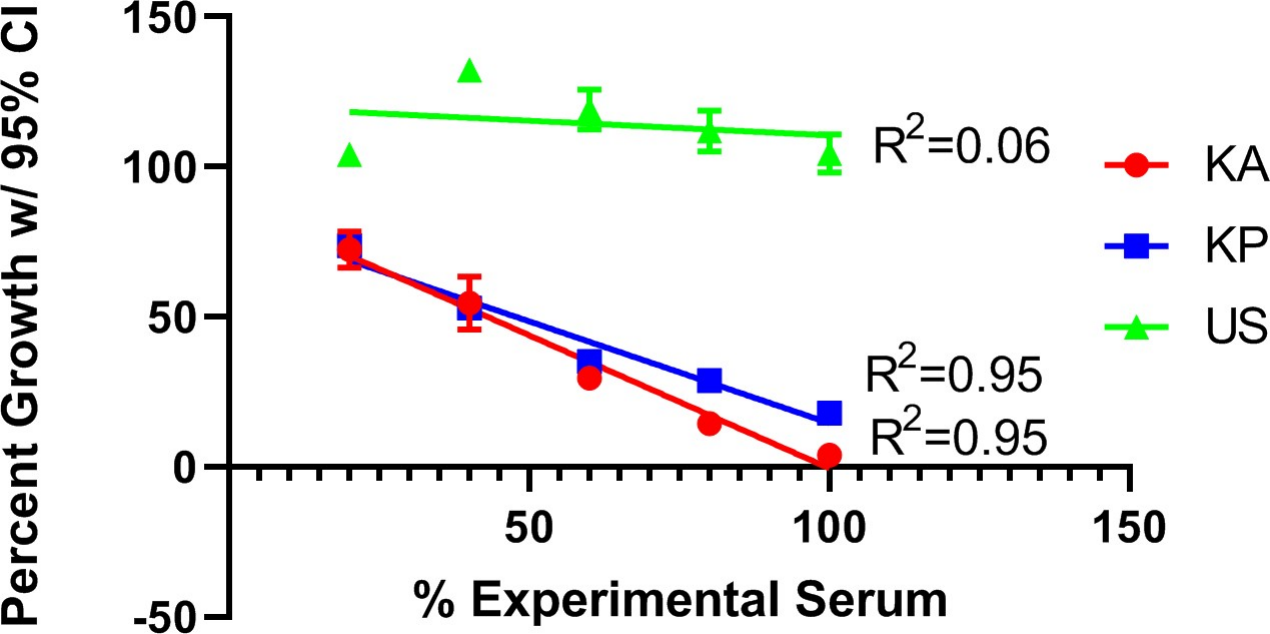
Plot of % parasite growth at 95% confidence index (CI) in various sera background vs. content of patient serum (Kenyan adult, Kenyan pediatric, US adult), as % of total serum used in each treatment (with commercial Sigma serum representing the remainder).

To observe the effects of components of the immune system on plasmodial growth, the 10% commercial human serum that was used to supplement the complete media was replaced with 10% human serum from Kenyan adults (KA), Kenyan children (KP), US adults (US), or a mix thereof. Initial attempts to use the treatment sera revealed reduced plasmodia growth in the Kenyan samples compared to the US samples and the KA and KP sera reproducibly inhibited plasmodia growth compared to the growth seen in the US serum or commercial Sigma serum in the absence of antimalarial drugs. Specifically, across two replicates, the growth observed in KA serum was 3.9% of growth in commercial serum, the growth observed in KP serum was 18.0% of growth in commercial serum, and the growth in US serum was comparable (104.5%) in commercial serum (Fig 6). This effect was so strong that the growth observed in these samples was too small to measure any additional inhibitory effects of drug treatment, so experiments using Kenyan sera were diluted with SHS to provide a measurable growth differential. Using a linear dilution series of the Kenya to commercial sera, there was a linear relationship between the amount of Sigma serum in the sample and the growth observed in Kenyan samples (R^2^ = 0.95 for both) (Fig 6). Dilution with Sigma serum, however, did not correlate well with the parasite growth observed from the US adult serum treatments (R^2^ = 0.06). Subsequently, treatment sera were diluted 1:1 in commercial Sigma serum in future experiments and all ART and DLAe-based treatments were at 9, 18, and 36 nM ART content.

Using the 1:1 serum dilutions in SHS, KA and KP sera inhibited parasite growth 71.5 and 73.5%, respectively, relative to US serum (Fig 6). Table 5 shows the IC50s of ART, DLAe, and *A*. *annua* tea treatments relative to the solvent control of each serum group. This representation separates the inhibitory effects of the serum and drug to test the null hypothesis that these two mechanisms of action are independent and that drug treatments will show similar inhibition regardless of serum background.

**Table 5:** Derived IC50s in nM ART of drugs and extracts under different serum backgrounds, biological N = 2.

IC50s (nM ART)	Treatment		
Serum	ART	DLAe	Tea
**UA**	11.3 (10.0, 12.6)	11.2 (11.2, *>36)	16.8 (18.1, 15.4)
**KA**	25.66 (*<9, 25.7)	32.6 (*, 32.6)	14.7 (16.1, 13.3)
**KP**	20.3 (15.6, 24,9)	15.9 (15.9, *>36)	16.0 (10.4, 21.6)

Parentheses describe the results of individual trials

* indicates the IC50 was either off the concentration range or crossed twice. ART, artemisinin; DLAe, dried-leaf *Artemisia* extract in dichloromethane; IC50, concentration of half-maximal inhibition; KA, Kenyan adult serum; KP, Kenyan pediatric serum; UA, US adult serum.

As a control, the effect of pure ART in each serum type was measured. Overall, the drug IC50s tend to be the lowest in the US group, followed by the KP groups, and highest in the KA groups. Notably, the calculated IC50s of the KA ART may have decreased if a replicate with an IC50 was beneath the concentration curve (<9nm) and had been included in the analysis (Table 5). In tea treatments, the degree of inhibition also is similar in all three sera with a putative IC50 of 14-17nM for all three treatments.

Within each serum group, there does not seem to be a replicable treatment pattern that is the most or least effective. DLAe may have produced higher IC50s and appeared less effective had two replicates that showed an IC50 above the curve (>36nM) been included in the analysis (Table 5). Overall, the results do not provide evidence of an interaction between components in malaria-exposed sera and anti-ring stage activity.

**Fig 7:**
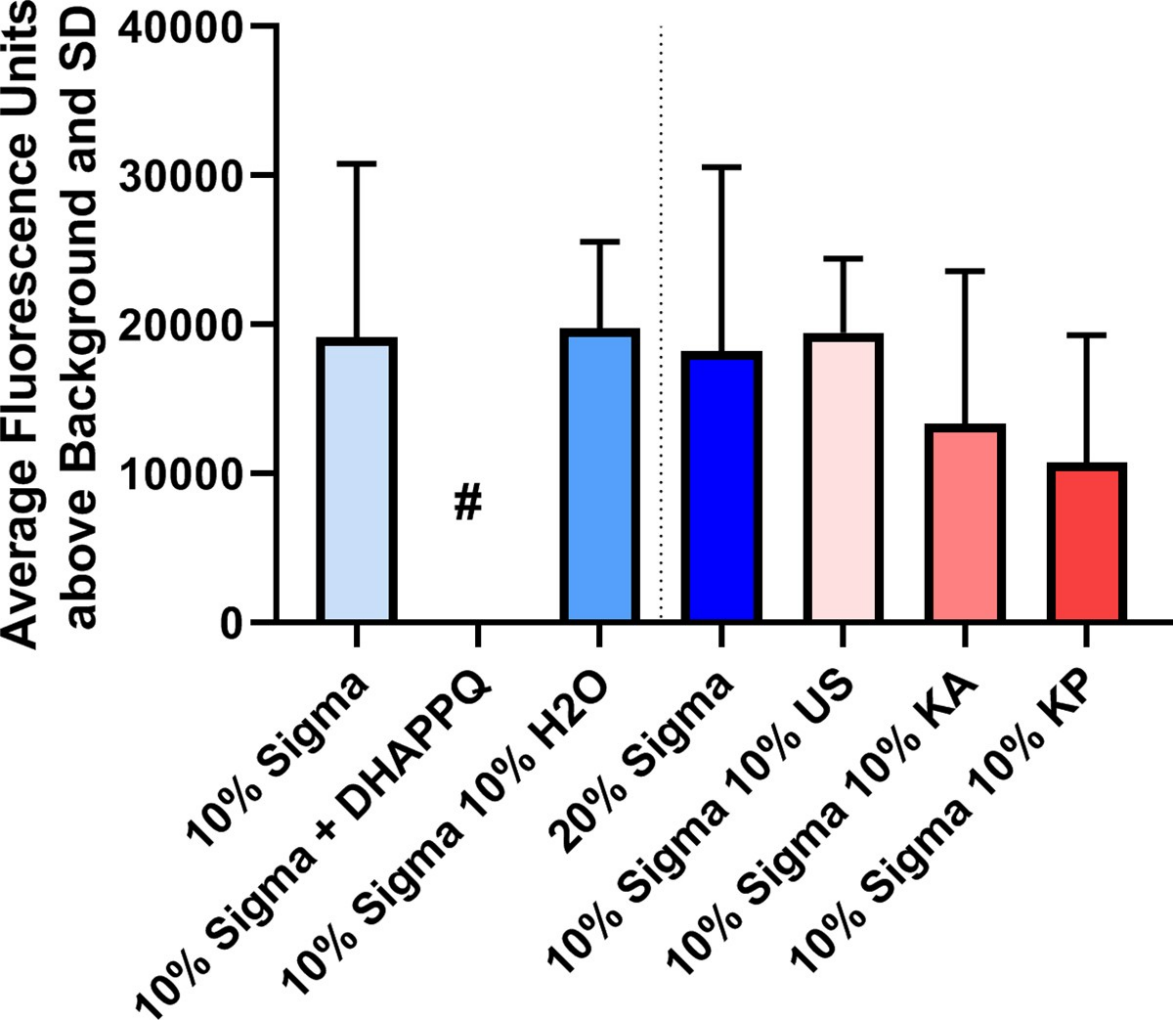
Averaged results of serum supplementation experiments. N = 2. DHAPPQ, dihydroartemisinin-piperaquine; H2O: Water; US: United States; # indicates value was undetectable.

Growth was inhibited in the presence of Kenyan serum, but it was still ambiguous whether inhibition was due to active inhibition from components of the serum or reduced growth from reduced-quality serum that did not support parasite growth. To determine if there was an active inhibitory component in the Kenyan sera, cultures of infected RBCs in 10% SHS were supplemented with various treatments (Fig 7). Compared to the unsupplemented 10% SHS group, supplementation with water, an additional 10% (to 20%), or 10% US serum did not substantially alter the average parasite growth. However, supplementation with either Kenyan serum significantly reduced overall parasite growth. Because the overall growth is less than even the water-supplemented control, it appeared that the Kenyan sera did indeed contain components that actively inhibited plasmodia growth.

## Discussion

The *A*. *annua* SAM cultivar was used in these experiments because of its favorable antimalarial properties against ART-sensitive *P*. *chabaudi* and resistant *P*. *yoelli* in murine models of malaria [15,16]. It was, therefore, hypothesized that synergistic interactions between the many compounds in *A*. *annua* with known antimalarial activity would explain its efficacy as an *in-vivo* antimalarial treatment. If this were true, it would follow that extracts of *A*. *annua* would exhibit considerably more potent *in-vitro* antimalarial activity against *P*. *falciparum* than ART and its derivatives. The *A*. *afra* SEN strain with similar phytochemical content, but negligible ART, would serve as an almost ART-free control, allowing for measurement of a non-ART effect.

In all the experiments, extracts of both *A*. *annua* and *A*. *afra* were effective in killing the ring stage of all the tested malaria parasite strains. Comparisons of 72-hr IC50s, however, did not yield conclusive evidence of antimalarial activity that could be explained by non-ART phytochemicals. As suggested by linear correlation of extract DW IC50 with phytochemical content, the IC50 of the extracts was strongly linked to ART content, while the link between flavonoid content and IC50s was tenuous at best. Of the tested extracts, only the dichloromethane extract (DLAe) showed a potentiation of inhibition activity beyond 1.5-fold, statistically significant at (*p* = 0.016; ɑ = 0.05/5 = 0.01). Increasing the predicted relative phytochemical to ART ratio by orders of magnitude through the use of *A*. *afra* instead of *A*. *annua* did not reduce the IC50. Finally, the apparent potentiation of ART seen in the dichloromethane extract of *A*. *annua* when used against the 3D7 strain was not recapitulated in the Cam3.II strain pair. This discrepancy could possibly be due to adaptation to antimalarial drugs and mechanisms in the more recently isolated Cam3.II strain pair compared to 3D7. This finding could also be interpreted as evidence against the slight enhancement of the extract’s antimalarial potential relative to ART as a true positive phenomenon.

The *A*. *afra* used in this experiment contained 2–3 orders of magnitude less ART/g DW of *A*. *annua* by plant mass. Although unusual for this species, the same cultivar grown also in Togo and from other harvest seasons also contained some ART, so it is assumed there is a slight biochemical difference, possibly from a field hybridization with *A*. *annua*. Regardless, the observed antimalarial activity correlated well with the ART content of SEN. In contrast, Mouton *et al*. did not observe any antimalarial activity in their preparations of *A*. *afra* up to 25 μg plant DW/mL [33]. In this study, the *A*. *afra* dichloromethane extract had an average IC50 of 178 μg plant DW/mL (Table 1), but it did not appreciably inhibit 3D7 at 25 μg plant DW/mL (4.2 nM ART) in our generated inhibition curves. This analysis supports the findings of Mouton *et al*. [33]. In comparison, the dichloromethane extract of *A*. *annua* was two to three orders of magnitude more potent by plant mass than *A*. *afra*. This finding is consistent with the relative difference in ART content and suggests that the *in-vitro* antimalarial potential of both *Artemisia* species was largely dependent upon ART content. A recent report corroborates the results of our study [34]. They used a different approach whereby they used an *A*. *annua* mutant with normal levels of phytochemicals but lacking ART. They also used 9:1 chloroform:ethanol solvent extracts of those plants to challenge *P*. *falciparum* Dd2 strain *in vitro*. They attributed all *in vitro* activity to only the presence of ART and not the presence of other antimalarial phytochemicals, e.g. flavonoids. In contrast, Snider and Weathers recently showed *in vitro* evidence that *A*. *afra* cultivars lacking detectable ART inhibited trophozoites [35]. Together these results suggest DLA *in vitro* drug responses are parasite stage sensitive.

Discrepancies between this and other studies may be attributed to a number of factors including parasite stage, i.e. trophozoite [20,31–35], vs. ring-stage (this study); mode of tea preparation, a boiling steep [33] a longer 15 or 60 min steep [20,31,32], or 10 min in boiled water (this study]; lyophilized tea [20,31]; solvent used to extract *Artemisia* sp. leaves for parasite treatment, e.g. hot water [20,31–34, this study] chloroform:ethanol [34], chloroform [33], dichloromethane (this study); method of parasite measurement, microscopic counting [32–34], lactate dehydrogenase [31,33] vs. SYBR Green assay [20,33,35, this study], etc. Unless such variables are fully controlled, the validity of any conclusion is challenging.

Because none of the conditions described in this report showed clear potentiation of extract antimalarial activity beyond ART content, the results did not compare with the efficacy of *A*. *annua* material as observed in Daddy et al [17]. Variables beyond those mentioned above, may have affected the outcome including loss of some components via the necessary filtration required for *in vitro* studies, strains of *P*. *falciparum* used, and method of parasite growth measurement. *A*. *annua* cultivars are inconsistently reported in the literature, and it appears that the plants used in the literature are grown in fields in diverse regions of the world, with no clear regional patterns. The strains of *P*. *falciparum* also varied, with the only consistent finding seeming to be that no potentiation is observed when the 3D7 strain is used [32,33]. The *P*. *falciparum* 3D7 strain is commonly used as a drug-sensitive control in experiments [41–43] and was chosen for this study because its ring stage was expected to be sensitive to the other active phytochemicals.

In this report, Cam3.II, a more recently isolated and ART drug-resistant strain, was also assayed for susceptibility to the methylene-chloride extracts; those extracts showed the most promise for potentiation in 3D7. This resistant strain was used to simulate the efficacy of DLAe therapy in a strain relevant to current resistance in Southeast Asia. However, this strain had a comparable response to DLA extracts (DLAe) and to ART treatments. Susceptibility of strain did not affect DLAe antimalarial performance vs. ART. It is possible that experimental design could influence outcome; IC50 determinations following 48-hour incubations with DLAe showed no potentiation [32,33] while 72-hour incubations seemed more likely to yield evidence of potentiation [20,31].

IC50s cannot effectively discern ART drug resistance [4,40]. There is a disconnect between the inhibitory and killing activity of ARTs in resistant strains of *P*. *falciparum* because these strains can survive treatment through entry into dormancy, which necessarily slows their growth [43,44]. Instead, ring-stage survival assays measured the -cidal activity of the extracts compared to ART and its derivatives. Following a six- or eight-hour incubation, DHA proved to be a significantly superior treatment to the ART-containing extracts. Equivalent antimalarial activity between ART and extracts with an equivalent ART concentration suggested that the short-term ring-stage killing activity of the extracts was solely due to its ART content. This finding is consistent with the findings that ART is the most abundant endoperoxide in *A*. *annua* [20] and that treatment with 700nM DHA in ring-stage survival assays results in lower survival rates than treatment with 700nM ART [45]. All treatments, however, were equally effective after a 24-hour incubation in high concentrations of ARTs and extracts, indicating that *in vitro* the extracts were equally effective, but at a slower rate than the pure ART drugs. In agreement with these findings, Sanz *et al*. (2012) determined that ART killed >99.9 percent of 3D7 parasites after 24-hour incubation at 320 nM ART [46].

These experiments used extracts of *A*. *annua*. No extract can precisely replicate the *per os* consumption of DLA used in prior rodent experiments [15,16,22]. Neither can use of 0.22 μm filtered tea compare with *per os* consumption of a tea infusion that still contains large amounts of fine particulates. The ingestion of insoluble plant material may represent a significant difference from these *in-vitro* experiments; indeed, supplementing oral ART with a plant-based mouse chow increased the uptake of ART in mice [22]. The removal of insoluble matter was necessary to perform *in-vitro* antimalarial experiments, but may have excluded important compounds present in the leaves and small stems that comprise DLA and material used in tea infusion preparations. In contrast, the use of aqueous *A*. *annua* infusions showed inhibitory activity beyond its ART content in other experiments [20,31].

It is also possible that the components present in *A*. *annua* were only active at higher concentrations and/or longer (>8 hrs) periods of incubation. Weak or slow activity could have been occluded by ART’s powerful, rapid antimalarial action in both the IC50 and RSA experiments. Because of ART’s brief elimination half-time of about 2 hours [47–49], residual antimalarial activity from persisting *A*. *annua* phytochemicals such as quercetin [50,51] could become medicinally significant after biological elimination of ART. There are, to our knowledge, no human pharmacokinetic data for ART delivery via DLA. The efficacy of non-ART phytochemicals hypothesis was challenged by measuring the IC50 of an *A*. *annua* ART-null mutant [34]. That mutant had an IC50 of 4,220 ng/mL, which was 271-fold higher than the wt *A*. *annua* extract. Similarly, our study used *A*. *afra*, which contained 0.23% the ART of *A*. *annua*, and had an IC50 of 1.18-fold higher than pure ART. Our low ART *A*. *afra* model, therefore, also contradicts the above “persisting phytochemical” hypothesis as measured against ring-stage parasites.

The discrepancy between *in-vitro* and *in-vivo* activities of the plant material suggests that there is an important interaction between the host and the DLA that potentiates therapeutic activity. Recently, Adderley *et al*. (2020) demonstrated the feasibility of targeting the host’s erythrocyte signaling pathways as an antiplasmodial strategy [52]. While this specific mechanism does not seem to be present in the DLAe material, a variety of possible mechanisms have been proposed. For example, in animal models, ingesting ART as DLA greatly improved its bioavailability [22]. Greater bioavailability of ART from DLA results from improved ART solubility from oils in *A*. *annua* [24], enhanced transport of ART across the intestinal mucosa [26], and inhibition by DLAe and some of its phytochemicals of liver cytochrome P450s, 2B6 and 3A4, that metabolize ART [30].

There is also evidence of a potential interaction between the host immune system and DLA in the difference in efficacy of DLA as a prophylactic against malaria in malaria-naïve vs exposed individuals. DLA was not an effective preventative measure against malaria for EU travelers [53], but *Artemisia* teas appeared to be prophylactic in those that have already had malaria [54,55]. The experiments in this paper probed for these interactions by examining the simultaneous effects of various human sera and DLA and may offer some insight into those prophylactic discrepancies.

Serum supplementation experiments consistently showed that serum from Kenyan patients with a history of malaria substantially reduced growth of the plasmodia parasite, while the US serum from malaria-naive patients did not. Furthermore, serum supplementation studies suggested that this phenomenon was not necessarily due to degraded serum quality, but due to some active inhibitory quality of the sera and plasmodia growth possibly was blocked by serum antibodies. In *in vitro* invasion assays, the Rh class of merozoite invasion proteins are implicated as a target of host IgGs [56]. Similar studies also implicated merozoite surface protein 1 (MSP1) [57,58] as a target of antibodies. *In vivo*, autoantibodies have activity against *P*. *falciparum*, and autoantibodies against phospholipids were associated with both asymptomatic malaria and lower parasitemia in symptomatic malaria [59]. Finally, maternal IgE levels have a potential protective effect on the fetus against malaria [60]. Our report is consistent with the finding that antibodies in patient sera can inhibit parasite reinvasion *in vitro* and apparently *in vivo*.

There was no clear evidence of an interaction between the antimalarial activity of sera and DLAe. At best, *A*. *annua* tea showed quantitatively higher levels of inhibition than pure ART in the Kenyan sera, but this pattern was not present in the DLAe, and the variability in these tests was too high to make any definitive conclusions. Given that antimalarial activity from the Kenyan sera and all antimalarial treatments occurred, evidence of an interaction between the two mechanisms was expected. Overall, neither direct antibody-phytochemical interaction nor antigen-phytochemical interaction appeared to be probitive mechanisms for antimalarial activity of ART or DLAe.

When discussing potential immune effects of DLAe, it is important to consider experimental limitations of using serum, which lacks white blood cells. Experiments only involved direct effects of non-living components present in serum, e.g. antibodies. While the experimental design provided evidence against DLAe interaction with these immunochemicals, it did not rule out interaction with the leukocytes that are part of the immune system. Evidence for medicinally relevant DLA interaction with T-helper cells exists in mouse models of treatment for leishmaniasis [29], but to our knowledge that effect is not well-studied with plasmodia. Further characterization of this interaction would require the use of studies performed in fresh plasma or continuous blood sampling of infected subjects. That could be performed either in patients with malaria or mouse malaria models.

DLA also has anti-inflammatory [28] and immunomodulatory [29] properties that could play a role in directing the host immune system to better combat the parasite. It is also conceivable that prodrugs in the milieu of DLA are activated by *in-vivo* metabolism. *In-vivo* transformation of prodrugs present in the material of plants into biologically active compounds has precedent [61] and would explain the discrepancy in the efficacies of *in-vitro* and *in-vivo* models. Future experiments should explore modulation of infected host immune systems, digestion systems, and DLA-ART human pharmacokinetics. Ideally, these experiments would use the blood, plasma, and serum of patients or healthy volunteers as model systems.

## Conclusions

DLA extracts and tea infusions of *A*. *annua* showed efficacy against the ring stage of multiple strains of *P*. *falciparum* and were indistinguishable in effect from ACT after 24 hours of *in vitro* treatment. *A*. *afra* showed a somewhat similar response once normalized to its ART content. None of the DLA extracts or tea infusions, however, was better than DHA, CoArtem^TM^ or ART controls. Overall, this set of experiments showed antimalarial activity of both the serum of malaria-exposed individuals and in DLAe. However, these activities appeared to be independent of one another. To characterize further potential DLA-immune interactions, antimalarial models that incorporate the living components of the immune system should be devised. Prior *in vivo* studies showed greater efficacy of DLA vs. control drugs, so this study and others indicated that *in vitro* approaches for measuring DLA efficacy are not always adequate to effectively assess the therapeutic potency of complex plant mixtures.

## Supporting information

S1 File (ZIP)

## Abbreviations

ACT: artemisinin combination therapy
ART: artemisinin
ASAQ: artesunate-amodiaquine
GCMS: Gas-chromatography/mass spectrometry
DCM: dichloromethane
DHA: dihydroartemisinin
DHAPPQ: dihydroartemisinin-piperaquine
DLA: dried-leaf *Artemisia*
DLAe: dried-leaf *Artemisia* extracts
DW: dry weight *Artemisia* material
FLV: flavonoids
IC50: 50% growth inhibition concentration
KA: Kenyan adult
KP: Kenyan pediatric
MeOH: methanol
RSA: ring-stage survival assay
SHS: Sigma human serum
US: US adult
